# Research progress on fermentation-produced plant-derived bioactive peptides

**DOI:** 10.3389/fphar.2024.1438947

**Published:** 2024-12-05

**Authors:** Lili Zhao, Xinhua Liu, Shuping Wang, Zhicheng Yin, Tianyue An, Jiayu Zhang, Ying Liu

**Affiliations:** ^1^ School of Traditional Chinese Medicine, Binzhou Medical University, Yantai, China; ^2^ College of Pharmaceutical Science, Shandong University of Traditional Chinese Medicine, Jinan, China

**Keywords:** fermentation, polypeptide, plant-derived bioactive peptides, microbial fermentation, bioactivity

## Abstract

With the advancement of biotechnology and the human pursuit of a healthy lifestyle, investigations on bioactive peptides (BAPs) have received increasing attention. Compared to proteins, BAPs have lower molecular weights and are more easily digested and absorbed by the human body, exhibiting various physiological functions. For instance, they can inhibit the angiotensin-converting enzyme, lower blood pressure, reduce cholesterol, and possess antioxidant, antimicrobial, and antiviral properties. BAPs are major functional food ingredients primarily derived from animals and plants. The latter are particularly favored due to their wide availability, low cost, and diverse bioactivities. In recent years, the research on plant-derived BAPs produced by microbial fermentation has progressed phenomenally. Consequently, this study provides a systematic overview and offers insights into the prospects of fermentation-synthesized plant-derived BAPs, aiming to provide a reference for their subsequent development and utilization.

## 1 Introduction

Bioactive peptides (BAPs) are low molecular weight (MW) protein degradation products that are easily digested and absorbed by the human body. They possess high physiological activity, acceptable safety, and low production costs. Compared to amino acids, peptide transport systems have advantages such as rapid transport, low energy consumption, and easy saturation. Consequently, the bioavailability of BAPs is higher than that of proteins and amino acids. They exhibit robust emulsifying properties, good hydration, and unique physiological functions unaffected by heating and pH changes (Zhao, 2018). The microbial fermentation-based preparation method of BAPs involves the hydrolysis of substrate proteins with proteases produced by growing microbes. Compared to others, this fermentation method is simple, cost-effective, and can be more easily industrialized, showing broad application prospects. This study provides a comprehensive review of the progress made in the research on microbial fermentation-based synthesis of plant-derived BAPs, offering an outlook on future research trends and aiming to provide a reference for their further study and applicability (Wang et al., 2024).

## 2 Preparation methods of plant-derived BAPs

**FIGURE 1:**
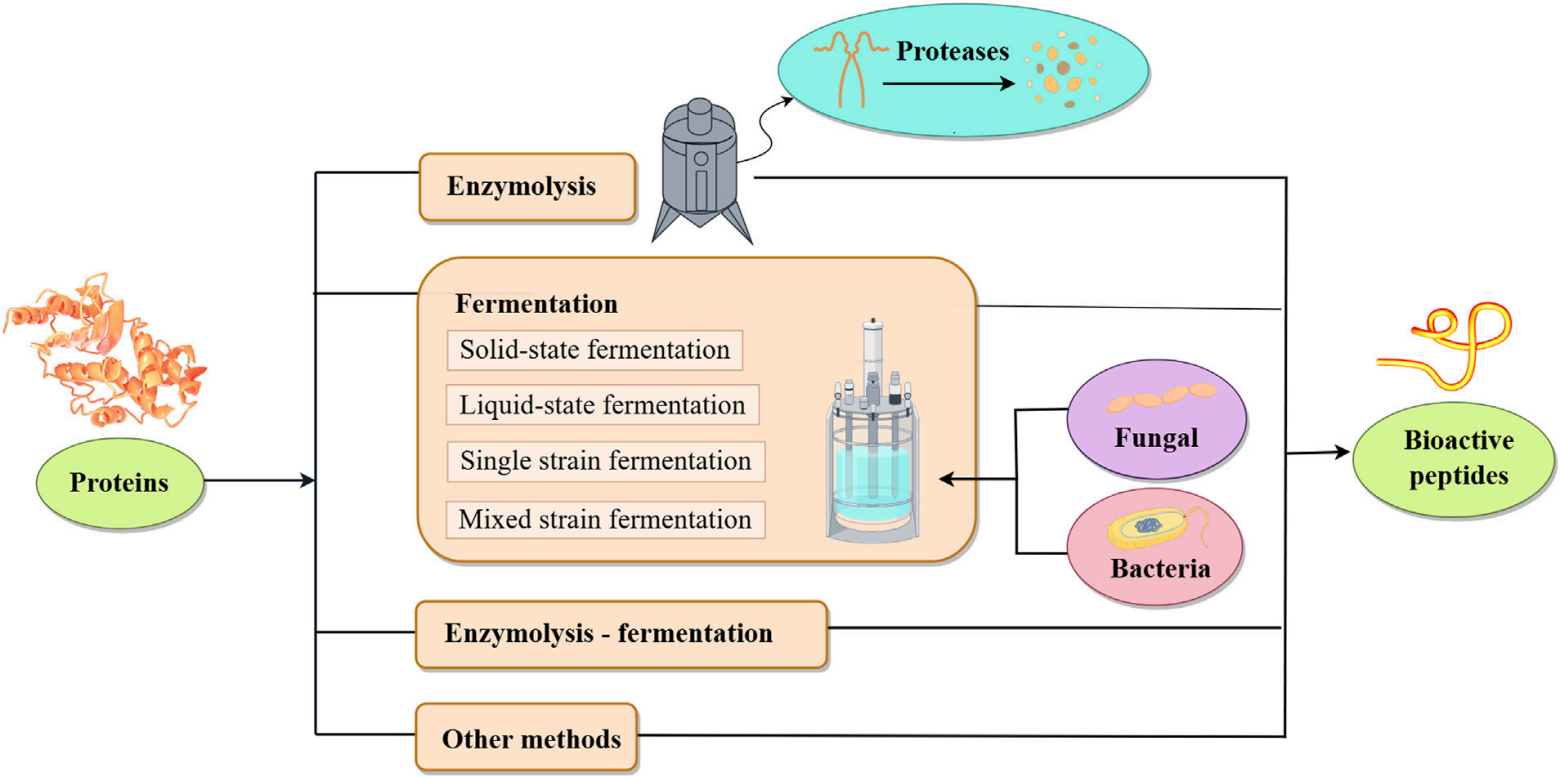
Various methods for preparing bioactive peptides from plants.

As shown in Figure 1, plant-derived BAPs are mainly prepared by direct extraction, enzymatic hydrolysis, biological fermentation and genetic engineering recombination.

### 2.1 Enzymatic hydrolysis (EH)

EH refers to the process of the hydrolysis of complex organic compounds into relatively simple molecules under the action of microbial enzymes. For example, using proteases can convert plant proteins into small MW peptides in a short time. Protease are rich in proteases, which are mainly divided into plant, animal and microbial proteases according to their sources. Among them, plant proteases account for a large proportion of soluble proteins in cells with high purity, such as soybean protease, maize protease, wheat protease, papain, etc. EH has lower production costs, mild reaction conditions, reasonable specificity, fewer byproducts, and fewer pollutants, thereby not harming the environment and is widely used in the food and pharmaceutical industries.

The optimal process for preparing angiotensin-converting enzyme (ACE)-inhibitory peptides by EH in sunflower seed meals through single-factor and orthogonal experiments was determined to be: solution pH of 8, enzyme addition at 7%, water bath temperature at 55°C, and hydrolysis time of 2 h obtaining an ACE inhibition rate of 85.49% ± 0.80% (La-Sheng et al., 2018). During the preparation of BAPs, EH has strict reaction conditions, and bitter peptides are produced. Moreover, using a single enzyme is poorly digested, time-consuming and the product has poor flavor. Therefore, in practice, a combination of multiple enzymes or a combination of EH with other techniques is usually adopted, which can not only reduce production costs but also improve the efficiency of EH.

### 2.2 Microbe-based fermentation method

Microbial fermentation is a method of producing BAPs by selecting suitable strains, utilizing proteases generated by microbial flora during their metabolic and physiological processes to hydrolyze substrate proteins, and then separating and purifying the fermentation products. Microbial cells can not only synthesize and secrete the corresponding BAPs through fermentation but also modify the functional groups of some bioactive peptides with their metabolites, thereby enhancing their bioactivity (Wang et al., 2024; Zhang et al., 2023).

#### 2.2.1 Solid-state fermentation (SSF)

SSF refers to the growth of microorganisms on an adequately moistened non-soluble medium in the absence or near absence of free-moving water, and is an aerobic process carried out on inert carriers or water-insoluble substrates. It is suitable for fungi with lower water requirements (Zhang et al., 2021). SSF was first applied to grain fermentation and has gained popularity due to its low cost, simple operation, mild reaction conditions, and suitability for large-scale production. The influencing factors and mechanisms of SSF in enhancing bioactive compounds in cereals and processing byproducts are reviewed (De-Villa et al., 2023). Microbial SSF can effectively promote the enhancement of BAPs and polypeptides in cereals and legume products, improving the antioxidant, anti-inflammation, anticancer, anti-diabetic, and ACE-inhibition effects of these bioactive components.

However, SSF relies on external O2 and is susceptible to contamination by a hybrid bacterium. It is less favorable for enhancing the yield of BAPs due to longer cultivation time and lower yield and productivity compared to liquid-state fermentation (LSF). Consequently, combining new technologies with SSF is more beneficial for enhancing BAP production.

In recent years, ultrasound (US) technology has been applied to overcome the limitations of SSF and increase the yield. For instance, it was utilized to treat *Bacillus amyloliquefaciens* during the SSF of soybean meal, which elevated the polypeptide yield (Wang et al., 2021). Moreover, the US enhances bacterial growth and their ability to produce proteases. The treated strains can increase the protein yield, protease activity, and polypeptide levels in the fermented soybean meal product. It can be inferred that US technology can effectively improve the yield of fermentation-derived plant-derived BAPs.

#### 2.2.2 Liquid state fermentation (LSF)

The most central or defining characteristic of LSF that differentiates it from SSF is that it uses a liquid substrate with over 95% moisture. LSF utilizes the symbiotic fermentation of fungi and bacteria, effectively leveraging their characteristics, which can result in more vigorous protease activity and faster fermentation rates. Compared with SSF, LSF has a shorter cycle, speedier cell proliferation, uniform cell development, and higher productivity, making it suitable for large-scale industrial production. However, its conditions are difficult to control, require higher parameters, and are prone to contamination. *B. subtilis* was used in the LSF of soybean meal, and the conditions were optimized through orthogonal experiments; the optimal conditions were determined to be an inoculum size of 10%, pH of 5, and cultivation at 40°C for 48 h (Liu et al., 2013).

#### 2.2.3 Single-strain fermentation

The single-strain fermentation method utilizes a single strain to ferment plant-derived proteins, thereby obtaining BAPs with specific physiological activities. This method has high controllability and specificity, making it easier to control the conditions and optimize the process. Fermentation broth prepared from *B. subtilis*, *Saccharomyces cerevisiae*, and *Lactobacillus* was used to conduct single-factor soybean meal fermentation experiments, with crude protein content as the main evaluation index, and the effects of fermentation temperature, material-water ratio, inoculum size, and fermentation time on the meal quality were investigated. Under optimal conditions, the crude protein content was elevated the most by 62.3% and the *Lactobacillus*-fermented soybean meal had the lowest crude protein content of 48.6%. However, the acidic aroma and pH could improve the palatability and attractiveness of the feed, achieving the purpose of improving the quality of the soybean meal (Fu et al., 2017). *B. subtilis* 1.0892 and *A. niger* 3324 were selected to ferment mung bean protein powder, and the corresponding fermentation conditions were optimized. The optimal conditions for *B. subtilis* 1.0892 were: inoculum size 11.61%, initial pH value 7.96, and protein powder 4.26%; those for *Aspergillus niger* 3324 were: inoculum size 10.51%, initial pH value 6.29, and protein powder 2.39% (Zhang, 2014).

Generally, single-strain fermentation is an effective preparation method with broad application prospects in plant-derived BAPs. However, it produces a relatively limited variety of BAPs, which may hinder the acquisition of diverse BAPs and restrict their applications in specific fields. To overcome this limitation, researchers have employed combinations of different strains for mixed cultivation to obtain a broader range and higher quantity of BAPs.

#### 2.2.4 Mixed cultivation

Mixed cultivation is a synergistic fermentation technique that utilizes two or more microorganisms, commonly bacteria and fungi. This mutual promotion is more conducive to the process, and the effect is superior to that of single-strain fermentation. The products of multi-strain complex fermentation may have better health-promoting effects than those of single-strain fermentation. Zhang. (2014) fermented mung bean protein powder with mixed strains (*B. subtilis* 1.0892 and *A. niger* 3324) and optimized the mixed culture process based on single strain fermentation. The results showed that mung bean polypeptide had strong antioxidant capacity, and the clearance rate of superoxide anion (O2-) and hydroxyl radical (HO) was 46.57% and 47.26%, respectively. The optimal process was achieved by mixing *A. niger* 3324 and *B. subtilis* 1.0892 in a ratio of 1:1.6, with an inoculum size of 10.27% and a protein powder of 4.32%. Due to the bitterness and odor values of the fermentation products of *B. subtilis* 1.0892 and *A. niger* 3324 single strains being relatively high, additional debittering and deodorization processes were required. However, the bitterness and odor of the mixed strain cultivation products were markedly reduced, indicating that mixed strains are more suitable than single strains for fermenting mung bean protein powder to prepare polypeptides and reduce process costs.

A mixed culture of *B. subtilis* and *Aspergillus oryzae* was utilized to ferment hazelnut meal; the process conditions were optimized through single-factor and orthogonal experiments, and the antioxidant properties and ACE inhibition rates of the products at different fermentation times were compared; the hazelnut peptides obtained demonstrated an O^2−^ scavenging rate of 98.75% when *A. oryzae* and *B. subtilis* were mixed in a 1:1 ratio, with a moisture content of 60%, an inoculum size of 15%, and 40°C, after 48 h of fermentation (Wang et al., 2018). *L. plantarum* B1-6, *R. oryzae*, or a combination of both were used for the SSF of whole-grain oats; the fermented oats had more bioactive and ACE-inhibitory peptides compared to unfermented oats; the degree of protein hydrolysis and polypeptide content were higher when the combination was utilized instead of *L. plantarum* alone (Wu et al., 2018). Orthogonal experiments were employed to determine the optimal fermentation conditions for cottonseed meal: initial moisture content of 50%, a total inoculum size of 3%, 30°C, and 96 h with A. oryzae and 24 h with *E. faecalis*; under these conditions, the crude and acid-soluble protein contents elevated; *Enterococcus faecalis* revealed an excellent detoxification effect; after adding *A. oryzae* for mixed cultivation, the detoxification rate and nutritional value were effectively improved, and the degradation effect of anti-nutritional factors was also enhanced (Wei et al., 2022). The above studies demonstrate that mixed cultivation can not only improve the efficiency and nutritional value of the products through the synergistic effects of different microorganisms but also broaden the product diversity and application range.

### 2.3 A composite EH–microbial fermentation method

Relying solely on microbial fermentation to produce plant-derived BAPs faces numerous challenges, such as certain byproducts that may be harmful to human health. Consequently, researchers have begun to explore new fermentation technologies, such as EH and membranes, to improve efficiency, product yield, and quality. For instance, a single EH can release high concentrations of BAPs with enhanced nutritional value, but their bitter taste leads to lower acceptability of their functional products. Microorganisms possess a complex enzymatic system for metabolism; utilizing them results in higher efficiency and bioactivity of the prepared peptides compared with traditional EH. Moreover, microbe-based metabolic fermentation can produce peptidases that hydrolyze bitter hydrophobic amino acids, thereby optimizing the taste and flavor of BAPs-based products (Wang et al., 2024).

### 2.4 Other methods

In addition to the above methods, direct extraction, chemical synthesis, and genetic engineering or recombination have also been used to produce BAPs. Direct extraction utilizes various separation and purification techniques to extract multiple BAPs from organisms directly. However, it has high processing and manufacturing costs, making it unsuitable for the extraction and separation of BAPs during large-scale production. It can easily cause environmental pollution, making it unsuitable for scaled-up production. Chemical synthesis, primarily the combined solid and liquid phase peptide synthesis created by Merrifield, is commonly used for the production of medium-length, highly pharmaceutically active peptides. However, it has high production costs, and the presence of residues and the occurrence of side reactions limit its large-scale production. Genetic engineering or recombination can only synthesize macromolecular peptides, and the expression of short peptides is challenging and inefficient. Therefore, compared with other methods, the microbial fermentation process for preparing BAPs is simpler, has a lower operating cost, and is more suitable for industrial production (Zhao, 2018; Wang et al., 2024).

## 3 Main microbial strains used for the fermentation-based synthesis of plant-derived BAPs

Various strains have been successfully applied to ferment and produce BAPs, including fungi such as yeasts and molds, as well as bacteria such as *lactic acid bacteria* and *Bacillus*.

### 3.1 Fungi

#### 3.1.1 Yeast-based fermentation

As one of the strains for the fermentation of plant-derived BAPs, yeast is currently the most widely used agent, commonly used yeast strains mainly include *Saccharomyces pastorianus*, *Pichia fermentans* and *baker’s yeast*, etc. These strains can produce different enzymes in the fermentation process, so as to effectively decompose proteins and generate peptides with various biological activities. The rapid propagation rate of yeast and the high requirement for dissolved oxygen are conducive to shortening the fermentation cycle. Zhao et al. (2022) used yeast to ferment wheat germ to study the effects of fermentation on the flavor characteristics and physiological activity of products, and optimized the fermentation process through single-factor experiments, providing technical and theoretical guidance for improving the processing utilization rate and product added value of wheat germ. However, the degree of aseptic requirements in the fermentation process is very strict, which increases the production cost. Mold fermentation may be more appropriate if needed to produce foods with specific flavor and nutritional value.

#### 3.1.2 Mold-based fermentation

Molds are an important class of natural carriers for BAP production. Mold-based fermentation is simple to operate, cost-effective, and easy to scale up for industrialization, thereby enhancing research on its use for the production of BAPs. Mold strains M-1 and M-2 with good enzyme production and activity, *B. subtilis* B-8, and *yeast* Y-3 were selected to conduct SSF experiments on soybean meal, the complex fermentation process conditions were optimized, and the changes in the contents of nutrients and anti-nutritional factors before and after SSF were analyzed; the results showed that using *B. subtilis* and *R. oryzae* in a ratio of 2:1, with a total inoculum of 10%, 40°C, a material-water ratio of 1:1.4, and fermentation for 96 h were the optimal conditions; post-fermentation, the peptide content in the product was markedly elevated; these results indicate that after the complex fermentation of soybean meal, the levels of nutritional components were markedly increased, the contents of anti-nutritional factors were remarkably reduced, and the nutritional quality was improved (Li X. G. et al., 2019). Mold fermentation and yeast fermentation have advantages to prepare active peptide, the specific selection depends on the characteristics of the desired peptide, production conditions and cost considerations.

### 3.2 Bacteria

#### 3.2.1 Lactic acid bacteria-based fermentation

Lactic acid bacteria strains are widely present in nature and in the human body. They are often used for fermentation-based synthesis of beneficial bioactive peptides. Their β-galactosidase can hydrolyze oligosaccharides during the fermentation of soybeans, reducing the beany flavor and flatulence. Whole wheat, soybean, barley, amaranth, and rye flours were fermented using lactic acid bacteria; the protease activity in the water-soluble extracts during fermentation was characterized using sodium dodecyl sulfate-polyacrylamide gel electrophoresis; post-fermentation, lunasin was quantified, purified, and identified in the water-soluble extracts using reversed-phase high-performance liquid chromatography (RH-HPLC) and nano liquid chromatography-electrospray ionization-mass spectrometry (LC-ESI-MS); compared with the control group (without lactic acid bacteria), fermentation enhanced the concentration of lunasin by 2–4-fold; the processes using *Lactic crispatus* SAL33 and *Lactic brevis* AM7 synthesized the highest concentrations of lunasin; *L. plantarum* is a common lactic acid bacterium that can proliferate rapidly and resist complex food matrix environments, showing a high potential for industrial applications; therefore, studying fermentation processes based on it is of great significance for the preparation of BAPs (Rizzello et al., 2012). *L. plantarum*-based fermentation of legumes was studied, the optimal conditions were determined, and the peptides with high α-amylase inhibitory activity were identified; the results showed that the 3.5–7.0 kDa components obtained after fermentation at 22°C for 3 h had inhibitory activity; after fermentation at 30°C for 3 days, the bean seeds released 3.5–7.0 kDa polypeptides, exhibiting the highest lipase and ACE-inhibitory activities; LC-MS/MS identified the soybean peptides with the highest activity to be INEGSLLLPH, FVVAEQAGNEEGFE, SGGGGGGVAGAATASR, GSGGGGGGGFGGPRR, INEGSLLLPH, GGYQGGGYGGNSGGGYGNRG, GGSGGGGGSSSGRRP, and GDTVTVEFDTFLSR (Jakubczyk et al., 2017). These observations enable new ideas and methods for finding potential BAPs that can inhibit obesity and metabolic syndrome.

#### 3.2.2 Bacillus-based fermentation

*Bacillus* is a class of microorganisms that can perform SSF or LSF, with the metabolites possessing various biological activities. *Bacillus*-fermented plant-derived BAPs can improve antioxidant capacity, enhance gastrointestinal function, increase nutrient absorption, boost immunity, reduce cholesterol levels, and elevate nutritional value and health benefits. *B. licheniformis* KN1G, *B. amyloliquefaciens* KN2G, and *B. subtilis* KN36D, KN2B, and KN36D were employed for the SSF of soybean meal; the results demonstrated that the antioxidant activity of water and methanol extracts from meal hydrolysates was markedly improved; the water-soluble extract of KN2B-fermented meal exhibited a 2,2-diphenyl-1-picrylhydrazyl (DPPH) free radical scavenging ability of 2.30 mg/mL, and the methanol-soluble extract of KN1G-fermented meal showed a rate of 0.51 mg/mL; simultaneously, unique peptides (HFDSEVVFF and VVDMNEGALFLPH) having antioxidant activity were identified from the fermented meal using LC-MS; moreover, the polypeptides were more diverse when KN36D was used (Kumari et al., 2023).

Fermentation using *Bacillus* not only enhances the antioxidant activity of BAPs but also promotes their hypotensive function. The bioactivity of fermented soybean meal using *Geobacillus stearothermophilus* was investigated; the bioactivity without high-pressure sterilization of the meal was markedly improved; the contents of polypeptides, crude, and soluble proteins elevated by 131.21%, 5.3%, and 15.52%, respectively; moreover, the reductive, DPPH, and ·OH scavenging abilities enhanced by 57.07%, 238.92%, and 368.26%, respectively; the ACE-inhibitory activity increased from 1.43% ± 0.83% to 26.89% ± 1.03%, while the activity of trypsin inhibitors declined by 74.05% (Wu et al., 2019).

## 4 Types of fermentation-produced plant-derived BAPs

**FIGURE 2:**
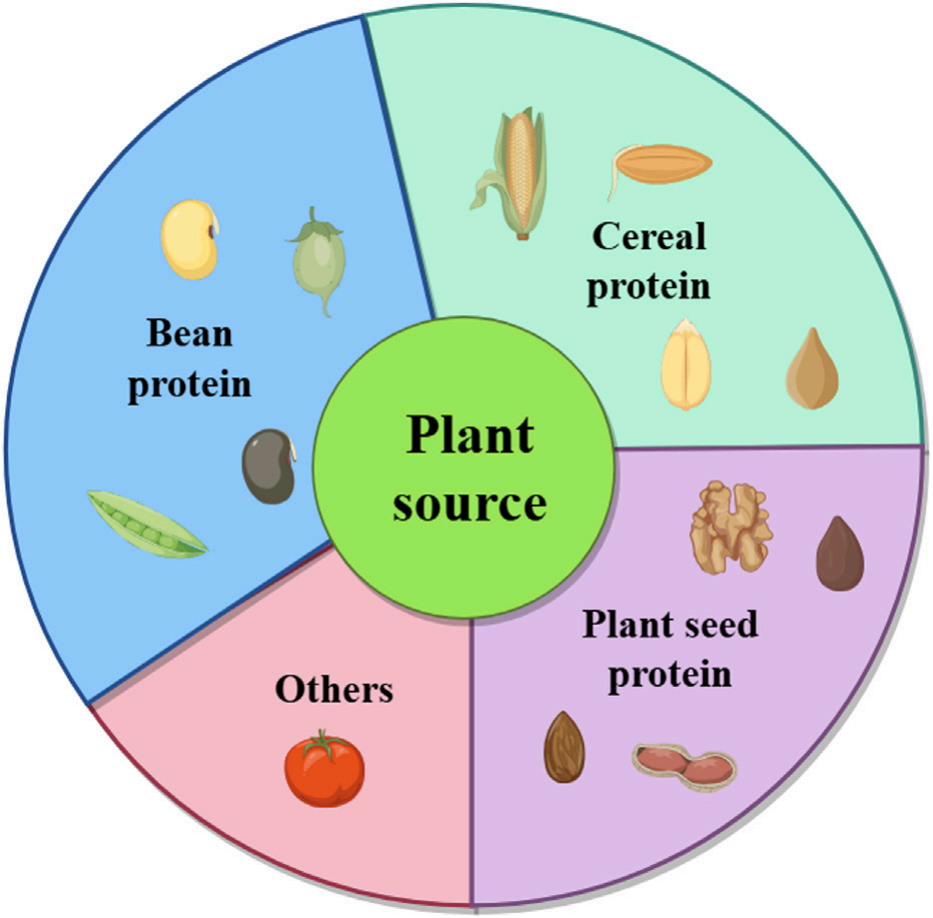
A variety of plant sources of BAPs.

Fermentation-synthesized plant-derived BAPs are diverse and include a wide range of peptides with specific bioactivities. As shown in Figure 2, they are typically derived from various plant materials, including proteins from legumes such as soybean, chickpea, mung bean, and pea; cereals such as corn, wheat, and sesame; and other plants. Table 1 presents a list of the common fermentation-produced plant-derived BAPs. Table 2 presents a list of the information about a study on the preparation of plant-derived BAPs by fermentation.

**TABLE 1:** Types of fermentation-synthesized plant-derived BAPs.

Source	Category	Bioactivity
Legume protein	Soybean peptides	Anti-oxidation, lowering blood pressure, immunoregulation, anti-inflammation, antimicrobial, anti-thrombotic, hypolipidemic, cholesterol removal, anti-tumor, anti-fatigue, and growth promotion
	Chickpea peptides	Anti-oxidation, lowering blood pressure, immunomodulation, anti-inflammation, antimicrobial, hypoglycemic, cholesterol removal, anti-tumor, and anti-fatigue
	Black bean peptides	Antioxidant, immunoregulation, hypolipidemic, anti-fatigue, sober
	Mung bean peptides	Antioxidant, lowering blood pressure, immunoregulation, cholesterol removal, anti-tumor, anti-fatigue, improving kidney function
	Pea peptides	Antioxidant, lowering blood pressure, immunoregulation, antimicrobial, anti-tumor, anti-fatigue
Cereal protein	Corn peptide	Lowering blood pressure, antioxidant, anti-fatigue, anti-cancer, liver protection
	Wheat peptide	Lowering blood pressure, antimicrobial, immunoregulation, anti-cancer, antioxidant, analgesic, anti-fatigue, agglutination and anti-agglutination, inhibition of apoptosis
	Rice peptide	Antioxidant, lowering blood pressure, immunoregulation, hypoglycemic, antimicrobial, anti-cancer, cholesterol removal, anti-aging, enhancement of food flavor
	Tartary buckwheat peptide	Antioxidant, cholesterol removal, lowering blood pressure, hypoglycemic, antimicrobial, anti-tumor
	Highland barley peptide	Antimicrobial, antioxidant, hypoglycemic, lowering blood pressure, sober
	Sesame peptide	Antioxidant, lowering blood pressure, antimicrobial
Plant seed protein	Cottonseed peptide	Antioxidant, immunoregulation, lowering blood pressure
	Rapeseed peptide	Antioxidant, anti-inflammatory, lowering blood pressure, anticoagulant, anti-AIDS, anti-cancer
	Peanut peptide	Antioxidant, lowering blood pressure, immunoregulation, anti-fatigue, antimicrobial, anti-cancer
	Almond peptide	Antioxidant, hypolipidemic, hypoglycemic, lowering blood pressure, anti-inflammatory, anti-cancer, antimicrobial
	Walnut peptide	Antioxidant, lowering blood pressure, anti-hyperuric acid, anti-cancer, antimicrobial, cardiovascular protection, immunoregulation, anti-fatigue, memory improvement
	Pine nut peptide	Antioxidant, lowering blood pressure, anti-tumor, hypolipidemic
	Prickly ash seed peptide	Antioxidant, lowering blood pressure, antimicrobial

**TABLE 2:** The information on studies on plant-derived BAPs produced by fermentation.

Matrix	Microorganisms employed	Process Parameters	Type of microbial fermentation	Effect	Reference
Soybean meal	*Bacillus amyloliquefaciens*	Cultivation at 37°C ± 0.5°C for 60 h	SSF under the ultrasound technique	Ultrasound technology can effectively increase the yield of plant-derived bioactive peptides during solid-state fermentation.	Wang et al. (2021)
	*B. subtilis*	Inoculum size 10%, pH of 5, and cultivation at 40°C for 48 h	LSF	The fermentation conditions for LSF of *Bacillus subtilis* were optimized.	Liu et al. (2013)
	*B. subtilis*	Water ratio of 1:1, Inoculum size 5%, and cultivation at 37°C for 48 h	Single-strain fermentation	Under the optimal process conditions of single bacteria fermentation, the crude protein content of the soybean meal samples fermented by *Bacillus subtilis* increased most.	Fu et al. (2017)
	*Saccharomyces cerevisiae*	Water ratio of 1:1, Inoculum size 5%, and cultivation at 31°C for 60 h			
	*Lactobacillus*	Water ratio of 1:1.2, Inoculum size 7%, and cultivation at 37°C for 72 h			
	*B. licheniformis* KN1G, *B. amyloliquefaciens* KN2G, and *B. subtilis* KN36D, KN2B, and KN36D	Cultivation at 37°C for 24 h	SSF	Unique peptides (HFDSEVVFF and VVDMNEGALFLPH) having antioxidant activity were identified from the fermented soybean meal and the polypeptides were more diverse when KN36D was used.	Kumari et al. (2023)
	*B. stearothermophilus*	Water ratio of 1:1, initial pH value 7, and cultivation for 72 h	Solid-state single-strain fermentation	Non-sterile SBM can be fermented by *B. stearothermophilus* at 55°C and is energy efficient and environmentally friendly.	Wu et al. (2019)
	*Mold strains* M-1 and M-2, *B. subtilis* B-8, and *yeast* Y-3	*B. subtilis* and *R. oryzae* in a ratio of 2:1, inoculum of 10%, material-water ratio of 1:1.4, and cultivation at 40°C for 96 h	SSF	The fermentation conditions of fermented soybean meal from *B. subtilis* and *R. oryzae* were optimized.	Li et al. (2019a)
Mung bean protein powder	*B. subtilis* 1.0892	Inoculum size 11.61%, initial pH value 7.96, and protein powder 4.26%	Single-strain fermentation	The fermentation conditions of fermented mung bean protein powder from *B. subtilis* 1.0892 were optimized.	Zhang. (2014)
	*A. niger* 3324	Inoculum size 10.51%, initial pH value 6.29, and protein powder 2.39%		The fermentation conditions of fermented mung bean protein powder from *Aspergillus niger* 3324 were optimized.	
	*B. subtilis* 1.0892 and *A. niger* 3324	*Aspergillus niger* 3324 and *Bacillus subtilis* 1.0892 in a ratio of 1:1.6, inoculum size of 10.27% and a protein powder of 4.32%	Mixed cultivation	The mixed strains are more suitable for fermenting mung bean protein powder than the single strains.	
Hazelnut meal	*B. subtilis* and *A. oryzae*	*A. oryzae* and *B. subtilis* in a ratio of 1:1, moisture content of 60%, inoculum size of 15%, and cultivation at 40°C for 48 h	Solid-state mixed cultivation fermentation	The fermentation conditions of solid-state mixed cultivation fermentation of hazelnut meal were optimized.	Wang et al. (2018)
Whole-grain oats	*L. plantarum* B1-6 and *R. oryzae*	Cultivation at 37°C for 0–72 h	Solid-state mixed cultivation fermentation	Fermented whole grain oats had more active peptides and ACE inhibitory peptides, and the combination of both strains had higher proteolytic content and polypeptide content.	Wu et al. (2018)
Cottonseed meal	*Bacillus subtilis*-1 and *S. cerevisiae*	Water ratio of 1:0.8, cultivation at 30°C for 72 h	Solid-state mixed cultivation fermentation	The optimal solid-state fermentation medium and optimal fermentation conditions for cottonseed meal fermentation were determined.	Liu et al. (2018)
	*A. Oryzae* and *Enterococcus faecalis*	Initial moisture content of 50%, inoculum size of 3%, 30°C, 96 h with A. oryzae and 24 h with *Enterococcus faecalis*	Mixed cultivation	The detoxification rate and nutrient content of cotton meal of mixed bacteria fermentation are higher.	Wei et al. (2022)
Wheat germ	*Yeast*	Yeast added 5 g/100 mL, pH of 4, material and liquid ratio 1:14 and cultivation for 48 h	Single-strain fermentation	The *yeast* prepared the flavor active peptide and optimized the fermentation conditions.	Zhao et al. (2022)
Whole wheat, soybean, barley, amaranth, and rye flours	*Lactic acid bacteria*	PH of 4, and cultivation at 30°C for 16 h	Single-strain fermentation	The fermentation process increased the concentration of lunasin by 2-to 4-fold, with the highest concentration of lunasin synthesized by SAL 33 and *L. brevis* AM 7.	Rizzello. (2012)
Bean seeds	*Lactobacillus plantarum*	Cultivation at 22°C for 3 h	Single-strain fermentation	The fermentation yielded a partial peptide with molecular weight of 3.5–7 kDa with α -amylase inhibitory activity.	Jakubczyk. (2017)
		Cultivation at 30°C for 3 days		The optimal fermentation conditions with the highest molecular weight of 3.5∼7.0 kDa, lipase or ACE inhibitory activity were determined.	
Italian legumes	*L. plantarum* C48 and *L. brevis* AM7	Cultivation at 30°C for 24 h	LSF	Yeast can significantly increase the content of antioxidants and protein lunasin-like polypeptides with physiological functions.	Rizzello et al. (2015)
Corn	*B. subtilis* Bls-45	The substrate–donor ratio of 8:1, the substrate concentration 6%, pH value 8; inoculum of 10%, and cultivation at 37°C for 46 h	Single-strain fermentation	The generated maize active peptide-glucose derivatives fully expressed the immune function of the maize active peptide.	Liu and Zhang. (2019)
Wheat bran	*Aspergillus oryzae*	Cultivation at 7°C overnight	LSF	The Kokumiγ -glutamyl peptide and volatile aromatic compounds were extracted, reducing the bitterness of the plant-based components.	Rodríguez Valerón et al. (2023)
Sesame meal	*Lactobacillus plantarum* and *Bacillus subtilis*	Inoculum of 8%, and cultivation at 30°C for 14 d	Solid-state anaerobic fermentation	The small peptides derived from solid-state fermented sesame powder exhibited vigorous antioxidant activity, and the antioxidant activity increases accompanied by decreasing the molecular weight of the peptide.	Xu et al. (2014)
Rice bran	*B. subtilis* 1389 and *A. niger* AS3.350	*B. subtilis* 1389 and *A. niger* AS3.350 in a ratio of 2:1, inoculum of 10%, rotation speed 200 r/min, and cultivation at 30°C for 24 h	Liquid-state mixed cultivation fermentation	The conditions for preparing ACE inhibitory peptide by liquid compound bacteria fermentation rice bran were optimized to improve the yield of ACE inhibitory peptide and the product inhibitory activity of ACE.	Li et al. (2013)
Coix seed rice bran	*B. subtilis*	Inoculum of 7%, initial pH value 7, and cultivation at 39°C for 24 h	Liquid-state single-strain fermentation	The fermentation conditions for polypeptide preparation by *B. subtilis* fermentation and the *in vitro* antioxidant activity were optimized.	Du et al. (2024)
Tomato waste	*B. subtilis* A14 h	Cultivation at 37°C for 24 h	LSF	Fermentation of tomato waste by *B. subtilis* can increase the relative number of aromatic amino acids and positively charged amino acids and affect the biological activity of peptide fragments.	Moayedi et al. (2017)
Okara	*Bacillus strains*	*B. subtilis* SNBS-3 and *B. amyloliquefaciens* 10 in a ratio of 1.5:1, inoculum of 1%, cultivation at 37°C for 40 h	SSF under the ultrasound technique	Optimizing ultrasonic technology can promote the stability and antioxidant activity of active peptide.	Xie et al. (2024)
Pomegranate juice	*Lactobacillus plantarum*	Cultivation at 37°C for 24 h	Single-strain fermentation	Both the ACE inhibitory activity and antioxidant capacity in pomegranate juice were significantly improved after fermentation.	Chen et al. (2023)
Tartary buckwheat	*Aspergillus oryzae, Aspergillus soy sauce, refined radiation hair mold, oligosporum*	Cultivation at 28°C for 7 d	SSF	*Aspergillus oryzae* was selected as the best bacterial strain for solid state fermentation.	Chen et al. (2018)
Chicken feather powder and okara	*Bacillus licheniformis mutant*	Okara and chicken feather powder in a ratio of 7:10, inoculum of 15%, cultivation at 37°C for 90 h	SSF	The fermentation conditions of solid-fermented chicken feather powder and okara were optimized using *Bacillus licheniformis* mutant and significantly increased the peptide yield and activity.	Tuly et al. (2022)
Astragalus, Epimedium, licorice, and other Chinese medicines	*Lactic acid bacteria*	Cultivation at 37°C for 48 h	Single-strain fermentation	The fermented Chinese herbal preparation could further promote the growth of chicks, reduce the feed-meat ratio, and lower production costs.	Li et al. (2020)

### 4.1 BAPs derived from legume proteins

Legumes such as soybeans, chickpeas, and peas are essential sources of proteins. Various methods, including EH, gastrointestinal digestion, and fermentation, can be used to prepare and purify a variety of BAPs from these proteins. These BAPs contribute to the prevention and treatment of chronic diseases, particularly inflammation-related disorders, obesity, and cardiovascular diseases. The functions of BAPs derived from various legumes in gastrointestinal health were reviewed, and some common legume varieties and their distribution were listed (Juárez-Chairez et al., 2022). Legume-derived polypeptides also possess multiple functions, such as lowering cholesterol, reducing thrombosis, and suppressing antioxidants.

#### 4.1.1 Soybean polypeptides

Soybean polypeptides possess various physiological functions, such as antioxidant, anti-hypertensive, anti-inflammation, anti-microbial, anti-thrombotic, anti-diabetic, cholesterol reduction, and immunoregulation effects (Singh et al., 2014). Soybean protein hydrolysates were analyzed using liquid chromatography time-of-flight mass spectrometry (LC-TOF-MS) combined with the technique of 2,4,6-trinitrobenzene sulfonic acid derivatization; a novel BAP, glycine-arginine, was identified, which promotes the expression of brain-derived neurotrophic factors and neurogenesis (Shimizu et al., 2018). Different *Bacillus* strains were utilized for the SSF of soybean meal, and the protein hydrolysates were extracted; by combining mass spectrometry techniques, bioinformatics prediction, and other methods, various BAPs were prepared and analyzed; different primary isolates produced various peptides, most of which exhibit varying antioxidant activities, providing an effective method for discovering and utilizing plant-derived BAPs (Kumari et al., 2023).

Lunasin is a soybean cotyledon-derived BAP that possesses antioxidant, anti-inflammation, anticancer, and anti-aging activities. *L. plantarum* C48 and *L. brevis* AM7 were used to ferment 19 Italian legumes, and Western blotting was performed on unfermented pod dough and yeast extracts using anti-lunasin antibodies to investigate the presence and physiological potential of lunasin-like polypeptides; ultimately nine lunasin-like polypeptides were identified; furthermore, during fermentation, lactic acid bacteria hydrolyzed the proteins, which can increase the quantity and activity of lunasin-like polypeptides (Rizzello et al., 2015). The soybean-derived BAP lunasin-encoding gene was employed to transform wheat via the *Agrobacterium* method, and its content in the L32-3, L32-6, and L33-1 transgenic lines was determined using enzyme-linked immunosorbent assay, WB, polymerase chain reaction, and ultra-performance liquid chromatography-mass spectrometry (UPLC-MS/MS) methods; additionally, the antiproliferative effect of lunasin on colorectal cancer HT-29 cells were detected, and the molecular structure and activity of lunasin were further investigated (Fan et al., 2020).

#### 4.1.2 Mung bean peptides

ACE-inhibitory peptides were isolated from mung bean dregs using EH and were further purified using ultrafiltration (UF), gel permeation chromatography (GPC), and RH-HPLC; three such novel peptides, FLVNPDDNENL, FLVNPDDNENLRII, and KDNVISEIPTEVLDL, were isolated (Li et al., 2014). Additionally, single and mixed cultivation of mung bean protein powder was conducted, and the fermentation conditions were further optimized (Zhang, 2014).

#### 4.1.3 Peptides from other legumes

Multifunctional cationic peptides were identified and characterized in the natto generated by *B. subtilis*-based fermentation; the natto extract contained cationic BAPs released from food-derived proteins, which were unable to induce hemolysis but could neutralize lipopolysaccharides and promote angiogenesis; this finding contributes to a deeper understanding of the mechanism of plant-derived BAPs and provides a new perspective for developing novel drugs with therapeutic potential (Taniguchi et al., 2019).

### 4.2 BAPs derived from cereal proteins

#### 4.2.1 Corn peptides

Corn-derived BAPs are products of corn protein hydrolysis, which can improve the defects of corn proteins, such as water insolubility. The current reported functional activities of corn BAPs primarily include sober, liver protection, anti-fatigue, and anti-oxidation. *B. subtilis* Bls-45 was used as a transformation platform strain for corn BAPs; the structure of corn BAP chains was modified through fermentation, and the corn BAP-glucose derivatives generated expressed the complete immune function of corn BAPs; single-factor and response surface optimization methods revealed that the optimal transformation was achieved when the substrate–donor ratio was 8:1, the substrate concentration was 6%, and the pH was 8; the optimal fermentation was achieved when the inoculation amount of Bls-45 was 10% and pH at 6, 37°C, and 46 h (Liu and Zhang, 2019).

#### 4.2.2 Wheat peptides

A study utilized two strains of *A. oryzae* for the miso fermentation of wheat bran and other cereal processing byproducts, as well as spent grains from beer production; Kokumi γ-glutamyl peptides and volatile aroma compounds were extracted by employing HPLC/MS-MS and headspace solid-phase micro-extraction gas-chromatography-mass spectrometry (HS-SPME-GC/MS), which reduced the bitterness of the plant-based ingredients (Rodríguez Valerón et al., 2023).

#### 4.2.3 Peptides derived from other cereals

Protein hydrolysates have been widely used as nutritional antioxidants, but their antioxidant activity remains unclear, for instance, small peptides. Sephadex G-15 chromatography and related animal experiments were applied to isolate and purify the small peptides from fermented sesame flour and study their antioxidant activity; their ability to scavenge DPPH and ·OH radicals, and their total reducing power was also measured; the small peptides exhibited vigorous antioxidant activity, which was inversely correlated with their MW (Xu et al., 2014). Additionally, the pretreatment process for preparing ACE inhibitory peptides from rice bran using mixed microbial fermentation was optimized; *B. subtilis 1389* and *A. niger AS3.350* were inoculated as the fermentation strains in a ratio of 2:1; post-pretreatment by the puffing method, the polypeptide yield and ACE inhibition rate of the product reached 38.86% and 64.48%, respectively, which were higher than those obtained by the starch removal pretreatment method (Li et al., 2013).

### 4.3 BAPs derived from plant seed proteins

Coix seed rice bran polypeptides were produced through fermentation using *B. subtilis*; the fermentation conditions were optimized using single-factor and orthogonal experiments; the *in vitro* antioxidant activity of the polypeptides was investigated; the polypeptides had good *in vitro* antioxidant activity (Du et al., 2024). UPLC/ESI-MS was employed to detect oligopeptides generated during the fermentation of cocoa beans from different origins and fermentation levels; a semi-quantitative analysis of the 35 identified low MW polypeptides was performed; the fermentation level and origin had a significant influence on the oligopeptide structure (Caligiani et al., 2016). The proteins and oligopeptides in fermented and unfermented cocoa beans were comprehensively analyzed; the peptide diversity was related to the fermentation method and degree but not to the origin (Kumari et al., 2018).

### 4.4 BAPs derived from other sources

BAPs are derived not only from legumes, cereals, and seeds but also from many herbs, melons, fruits, and vegetables. A fermentation study conducted on tartary buckwheat using *lactic acid bacteria* identified six polypeptides (DVWY, FDART, FQ, VAE, VVG, WTFR, and tyrosine) through LC-MS and Edman degradation; fermented tartary buckwheat sprouts could produce antihypertensive peptides with elevated contents of preexisting bioactive compounds, such as 4aminobutyric acid and tyrosine, providing a new approach for the treatment of hypertension (Koyama et al., 2013). Tomato waste was fermented using *B. subtilis*, and the influence of amino acid composition and molecular mass distribution on the ACE inhibitory and antioxidant activities of the polypeptides was investigated (Moayedi et al., 2017). A subsequent study separated and purified the antioxidant and ACE-inhibitory peptide mixtures obtained through different chromatographic steps and identified specific components with antioxidant and ACE-inhibitory activities using nano-liquid chromatography-mass spectrometry; the fermentation of tomato byproducts by *B. subtilis* could yield polypeptides with high added value (Moayedi et al., 2018).

## 5 Effects of microbial fermentation on physiological activity of BAPs

**FIGURE 3:**
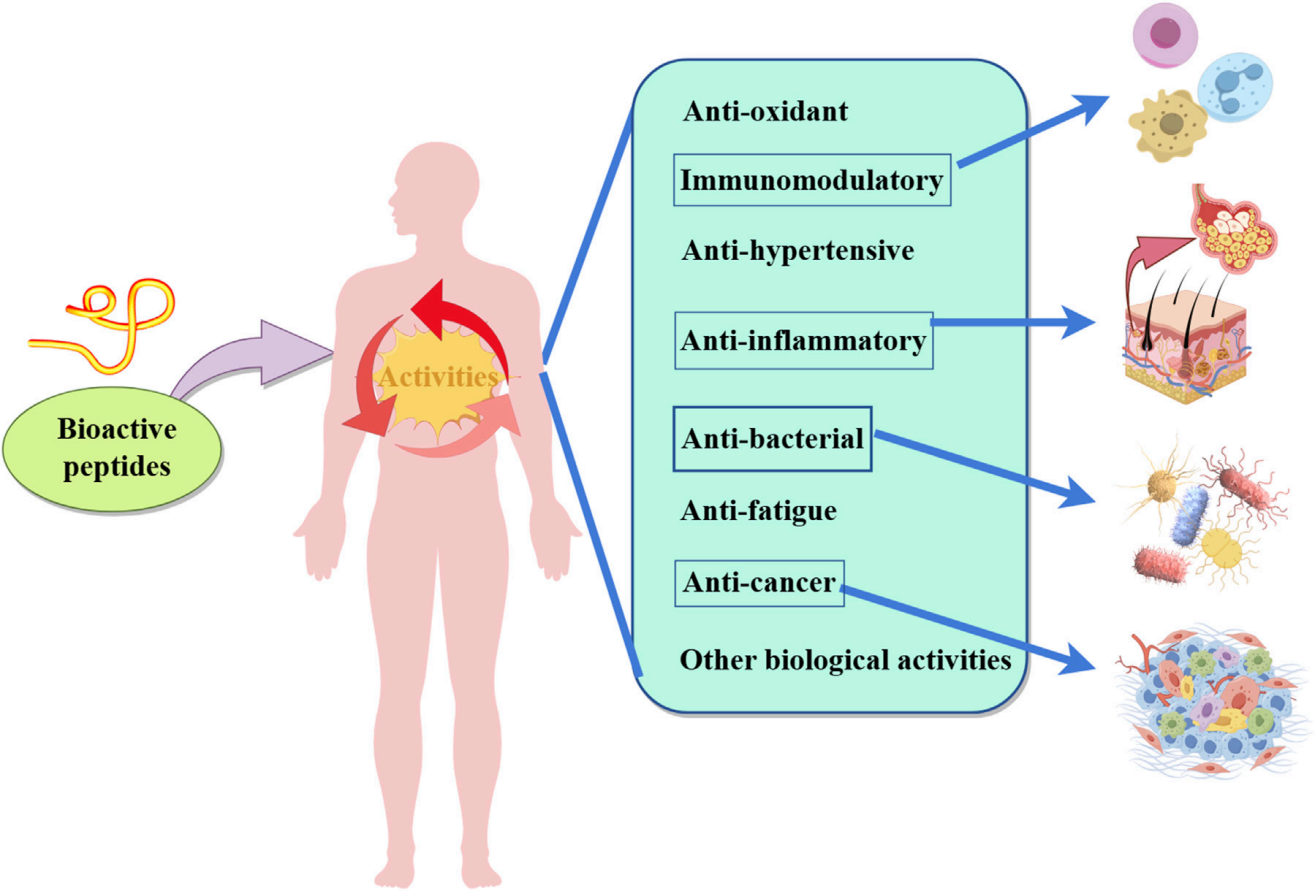
Multiple physiological functions of Plant-derived BAPs.

As shown in Figure 3, Plant-derived BAPs have a variety of physiologically active peptides, and microbial fermentation can convert certain compounds into metabolites with biological activity, effectively increasing the yield and activity of plant-derived BAPs. The BAPs in fermented cucumbers were identified and quantified using infrared matrix-assisted laser desorption electrospray ionization mass spectrometry (IR-MALDESI-MS) and liquid chromatography-triple quadrupole mass spectrometry (LC-QQQ-MS); the fermented cucumbers contained three ACE inhibitory peptides: Ile-Pro-Pro (0.42–0.49 mg/kg), Leu-Pro-Pro (0.30–0.33 mg/kg), and Val-Pro-Pro (0.32–0.35 mg/kg). Compared to acidified cucumbers, the content of the ACE-inhibitory peptide Lys-Pro (0.93–1.5 mg/kg) in fermented cucumbers elevated by 3–5-fold; these results indicate that *lactic acid bacteria*-based fermentation can enhance the content and biological activity of BAPs in vegetables (Fideler et al., 2019).

### 5.1 Effect of fermentation on antioxidant activity of BAPs

Antioxidant activity generally refers to a substance or compound that scavenge free radicals, protecting cells from oxidative damage. The antioxidant products generated through fermentation can help reduce the risk of oxidative damage-related diseases. Microbial fermentation can affect the release of antioxidant peptides, elevate the metabolic activities of phenols and flavonoids, and enhance the contents and capacities of antioxidants (Verni et al., 2019). The effects of fermentation on bioactive components and antioxidant activity have been reviewed (Zhao et al., 2021). Fermentation can promote the breakdown of plant cell walls and facilitate the release or production of various antioxidants. Additionally, it can also promote the generation of antioxidant polysaccharides and peptides while enhancing their activity.

Compared to unfermented soybeans, fermented ones exhibited stronger *in vitro* free and O-2 scavenging abilities, which may be related to changes in polyphenol content and the digestibility of soybeans after fermentation (Dimidi et al., 2019). Antioxidant peptides were prepared using US-assisted fermentation technology, and optimization, stability analysis, and functional studies were conducted (Xie et al., 2024). After US-treatment, the content of fermentation-produced antioxidant peptides and their DPPH free radical scavenging rate reached the highest. Furthermore, US-assisted technology can markedly enhance the stability and activity of antioxidant peptides.

### 5.2 Effect of fermentation on the hypotensive activity of BAPs

Hypotensive activity is the anti-hypertensive efficacy of a compound or drug. Drugs with anti-hypertensive activity can have a therapeutic effect on physical diseases such as hypertension, helping to reduce blood pressure and reduce the burden of the body. Microbial fermentation can regulate the biotransformation of plant-derived phenolics, enhance the ACE inhibitory activity of plant-based foods, and promote the blood pressure-lowering effect of phenolics. *L. plantarum* was utilized to ferment pomegranate juice, markedly improving its ACE inhibitory activity and antioxidant capacity (Chen et al., 2023). This study was the first to reveal the relationship between ACE inhibitory activity and the biotransformation of phenolics in fermented pomegranate juice, providing a new approach to enhance the ACE inhibitory potential of plant-based foods.

A combination of new technologies and fermentation methods can further enhance the hypotensive effect of fermented polypeptides. Ultra-high pressure processing (UHPP) was used to study the polypeptide concentration, ACE inhibitory and antioxidant activities, and physicochemical properties of fermented milk produced by *Lactobacillus delbrueckii* QS306; peptides were effectively identified in the fermentation products, and the effect of UHPP on enhancing the ACE inhibitory activity of the products was ascertained through peptidomics; UHPP could markedly increase the concentration of polypeptides and volatile aromatic compounds in the fermented milk, as well as the ACE inhibition and antioxidant activities; this method enables the discovery and identification of new ACE inhibitory peptides and offers novel ideas and a practical basis for the production of fermentation-obtained plant-derived BAPs and the improvement of nutritional functions (Wu et al., 2022; Wu et al., 2023).

### 5.3 Effect of fermentation on the hypoglycemic activity of BAPS

Type 2 diabetes mellitus (T2DM) is a chronic disease caused by insufficient insulin utilization or efficiency, which is often accompanied by many complications, such as a series of cardiovascular and cerebrovascular diseases caused by abnormal lipid metabolism. Therefore, hypoglycemic activity studies are crucial for physiological health. Asians have a lower incidence of T2DM compared to Westerners, which may be due to the unique Asian habit of consuming fermented soybean products, among others. Phytoestrogens and polypeptides in fermented soybean foods may help prevent and delay T2DM. The effects of fermented soybean products in preventing and treating T2DM were reviewed (Kwon et al., 2010). Consuming soybean protein containing isoflavones can control blood glucose levels and reduce insulin resistance, while consuming fermented products can suppress insulin resistance and improve insulin secretion. Although human intervention trials have not verified this conclusion, evidence suggests that fermented soybean products are more beneficial in preventing or delaying T2DM compared to unfermented ones.

### 5.4 Effect of fermentation on the antimicrobial activity of BAPs

Antimicrobial activity usually refers to the ability of antimicrobial agents to inhibit or kill pathogenic microorganisms. The study of antibacterial activity is of great significance to improve the treatment effect, reduce the generation of drug resistance, and guarantee the health of patients. Antimicrobial peptides derived from plants have low MW, exhibit vigorous antimicrobial activity, and good water solubility. SSF of tartary buckwheat by *A. oryzae* was used to obtain the antimicrobial peptide “Gln-Pro-Glu-Asp-Phe-Arg;” the Plackett-Burman test and response surface methodology were applied to optimize the process on the basis of which, the preservation effect of the antimicrobial peptides was investigated; the shelf life of tilapia fillets treated with the peptides was extended by 4 days, and their antimicrobial effect was better than that of nisin; this finding provides a theoretical basis and technical support for the preparation and large-scale production of antimicrobial peptides (Chen et al., 2018).

### 5.5 Effects of fermentation on other bioactivities

The viable counting method was utilized to detect *L. plantarum* NDC75017, and it’s *in vitro* cholesterol-reducing ability was determined using the o-phthalaldehyde method; the results showed that NDC75017 has good environmental tolerance and *in vitro* cholesterol-lowering effects, and this strain can be used as a potential probiotic for cholesterol elimination (Wang et al., 2013). Consequently, it is speculated that this effect of BAPs can be further enhanced after fermentation by NDC75017. Various BAPs with cholesterol-lowering effects were obtained from the enzymatic hydrolysate of glycinin; rats fed with soybean peptides demonstrated a significant reduction in their serum cholesterol concentration, along with a marked increase in the fecal excretion of cholesterol; the reason may be that soybean peptides can inhibit the absorption of cholesterol by the intestines, stimulate the secretion of thyroid hormone, promote the conversion of bile acids and their excretion, thereby enhancing the fecal excretion of cholesterol to lower its levels (Takenaka et al., 2000).

## 6 Separation and purification of plant-derived BAPs from the fermentation broth

The separation and purification of BAPs are the foundation for investigating their biological activities and structural identification, as crude BAP products contain a large number of impurities and exhibit low biological activity. Traditional methods are time-consuming, inefficient, and yield poor quality products. Therefore, new biotechnological approaches are urgently needed. Currently, the most widely used methods include UF, GFC, ion exchange chromatography (IEC), HPLC, or a combination of these (Li et al., 2022).

### 6.1 UF

UF is a membrane-based separation technology driven by pressure, which separates polypeptides of different MWs through the microporous structure of a semi-permeable membrane and enables the determination of their biological activity. This technology is simple to operate and ensures the integrity of substances. However, as the running time increases, concentration polarization, membrane pore blockage, and gel layer formation can reduce membrane flux. Currently, the most widely used membrane technology for the separation and purification of active peptides is extensively applied in various fields, such as pharmaceuticals and healthcare, food processing, and sewage treatment (Song et al., 2019). Fermentation-based soybean meal protein was produced using the Y4 strain with the highest protease activity; after preliminary treatment of the fermentation broth, it was subjected to graded filtration through ultrafiltration membranes with MW cut-offs of 10,000, 1,000, and 500 Da, obtaining three respective polypeptide mixture components: F1, F2, and F3; subsequently, their vascular activity and ACE inhibition rates were investigated (Cui et al., 2018).

### 6.2 GFC

GFC, also known as molecular-exclusion chromatography or gel chromatography, is a common method used for separating, purifying, and detecting the MW distribution and amino acid content of polypeptides. As the BAP products require high purity, GFC is often used for their preliminary separation and ion exchange chromatography for their purification (Lazcano-Pérez et al., 2012). WGH-80% dextran Sephadex G-15S gel was utilized to separate wheat gluten protein hydrolysates; it was found that the second component exhibited the most robust activity in promoting yeast growth and metabolism; among these, small peptides such as AQP, ENG, LIR, SSR, LIM, LIPPY, and PPY played essential roles (Zhou et al., 2017).

### 6.3 HPLC

HPLC technology is employed for the separation and purification of polypeptides based on the differences in hydrophobicity between them and the stationary phase. When the polarity of the mobile phase is lower than that of the stationary phase, it is referred to as normal-phase HPLC, whereas the opposite is known as RH-HPLC. RH-HPLC offers high resolution, excellent separation, and strong adaptability, making it the most commonly used method for the separation and purification of BAPs (Yang et al., 2023).

### 6.4 IEC

IEC separates and purifies polypeptides by exploiting their interaction with ions, where the charges carried by the polypeptides bind to the opposite charges of the ion exchanger. For instance, a 17-amino acid peptide was purified using IEC (Boudesocque et al., 2017). IEC alone is rarely used in current applications for polypeptide separation and purification. It is commonly combined with RH-HPLC and GFC to leverage the complementary advantages of multiple separation techniques, which is more beneficial for the separation and purification of complex samples. Polypeptides were separated using a combination of an anion exchange column and RP-HPLC, and fractions with robust antioxidant activity were isolated based on the differences in charge and hydrophobicity (Xing et al., 2016).

## 7 Current applications of fermentation-produced plant-derived BAPs

BAPs have a significant nutritional value. For instance, oligopeptides can stimulate insulin secretion, while longer peptides enhance protein synthesis and the absorption of phosphopeptides from minerals and casein (Zhao, 2018).

### 7.1 Application value in the food industry

With an aging population, the growing number of sub-healthy people, and the rising incidence of chronic diseases in China, people’s awareness of healthcare is also increasing. Thus, the demand for natural, nutritious, and functional foods is becoming more evident. In recent years, BAPs have become an important bioactive factor with excellent development prospects in China’s food industry. They can protect cell structures and regulate human physiological functions (Guo et al., 2019; Mackie, 2020; Li Z. H. et al., 2019).

Additionally, they possess various functions such as antimicrobial, antioxidant, hypotensive, and immunoregulative effects, making them essential active components in functional foods, condiments, and medicines (Iwaniak et al., 2019). Moreover, peptides have small molecular sizes and fast absorption rates, allowing them to be directly absorbed by the human intestine. Consequently, they are often used as excellent nutritional supplements for special population groups such as infants, young children, and the elderly. Fermented foods have unique flavors and marked probiotic functions, making them a natural treasure trove of BAPs. Currently, various functional peptides with excellent nutritional value, flavoring characteristics, and biological activities have been discovered in fermented foods (Zhao, 2018).

### 7.2 Application value in the feed industry

Fermentation-obtained plant proteins have a high nutritional value and low cost, making them a potential alternative to expensive fish meal in fisheries, reducing production costs. Moreover, compared to fermentation-synthesized plant proteins, BAPs are more easily absorbed by the body, can partially replace antibiotics, promote the absorption of minerals, improve feed quality and flavor, and meet the nutritional needs of poultry and livestock. *A Bacillus* mutant was used to convert chicken feather meal and bean dregs into BAPs through SSF, optimizing the inoculation conditions and formulation of the mixed waste, markedly elevating the yield and activity of the peptides; in addition to *Bacillus*, the application of *lactic acid bacteria*-fermented traditional Chinese medicine has also shown promising results in poultry and livestock farming (Tuly et al., 2022). *Lactic acid bacteria* were employed to ferment the feed containing Astragalus, Epimedium, licorice, and other Chinese medicines; the results showed that the fermented Chinese herbal preparation could further promote the growth of chicks, reduce the feed–meat ratio, and lower production costs (Li et al., 2020). The medium and culture conditions were optimized using single-factor, orthogonal optimization, and response surface optimization experiments when preparing cottonseed meal oligopeptides by SSF with a compound strain; the antioxidant and immune activities and the effects on the growth performance and the expression of peptide transporter PepT1 in the small intestines of mice were evaluated; the fermented cottonseed meal rich in oligopeptides had good applicability in the feeding of yellow-feathered broilers (Liu et al., 2018).

### 7.3 Application value in the cosmetics industry

Plants, especially medicinal plants, contain a wide variety of bioactive compounds, which have broad application prospects in cosmetics. However, the effective bioactive components in plants are relatively low, and some may even have toxic side effects; traditional extraction methods have lesser efficiency, which seriously limits their application in the raw materials of cosmetics (Galkanda-Arachchige et al., 2019). Microbial fermentation can promote the release and enrichment of bioactive components or transform them into novel products with more substantial influence and reduced toxic side effects, effectively avoiding the drawbacks of traditional extraction methods. Additionally, plant-based fermentation-derived bioactive compounds have multiple effects such as antimicrobial, antioxidant, anti-inflammation, whitening, and collagen regeneration promotive activities, which have broad application prospects in cosmetic products.

## 8 Conclusion and prospects

With people’s pursuit of a healthy lifestyle and preference for natural foods, research and application of fermentation-obtained plant-derived BAPs have gradually gained widespread attention. Plant-derived BAPs have low MWs and can be absorbed and utilized by the body in their intact form. Furthermore, microbial fermentation can markedly influence the BAPs, effectively enhancing their physiological activity and safety while reducing production costs and providing strong support for the development and application of these BAPs. This study summarizes the latest progress in research on fermentation-synthesized plant-derived BAPs, including their type, preparation, microbial strains, and their physiological activity. By selecting different plant raw materials and microbial strains for fermentation, various peptides with biological activities such as antioxidant, antiinflammation, hypoglycemic, and hypotensive properties have been successfully prepared. They not only retained the nutritional value of plant proteins but also produced more bioactive compounds during the fermentation process, which are beneficial to human health.

However, some challenges regarding the investigation of fermentation-produced plant-derived BAPs still exist. For instance, the absorption, transportation, metabolic mechanisms, and physiological significance of BAPs in the human body are still unclear. Further research is needed to investigate the structure and function of peptides deeply, reveal the mechanism underlying their bioactivity, and translate the results into practically applicable products that can meet market demands. Future research on fermentation-synthesized plant-derived BAPs should deepen. On the one hand, researchers should continue to investigate the combinations of different plant raw materials and microbial strains to prepare novel BAPs. On the other hand, they can improve the yield and activity of peptides by modifying and optimizing the microbial strains applied for fermentation and employing new technologies as auxiliaries. In summary, as natural bioactive compounds with broad application prospects, fermentation-produced plant-derived BAPs are expected to receive more research attention and exploration in the future, making greater contributions to human health.
